# Supplementary material for: Calcitonin receptor expression in medullary thyroid carcinoma
Source: PeerJ. 2017 Sep 13;5:e3778. doi: 10.7717/peerj.3778 (PMC5600720; doi:10.7717/peerj.3778)
Supplement: Supplemental Information 1 [file peerj-05-3778-s001.docx]

Supplementary Table: Summary of the clinical, pathological and molecular data of the MTC cases

|  | **MTC** |
| --- | --- |
| **Gender (n=54) ***  **Female**  **Male** | 29 (53.7 %)  25 (46.3%) |
| **Age (years) ***  **(n=54; mean ± S.D.)** | 53.6 (± 14.1) |
| **Age (n=54) ***  **<45 years**  **≥45 years** | 14 (25.9%)  40 (74.1%) |
| **Tumor size (cm) ***  **(n=38; mean ± S.D.)** | 2.6 (± 1.7) |
| **Tumor size (n=38) ***  **<2 cm**  **≥2 cm** | 18 (47.4%)  20 (52.6%) |
| **Stroma (n=71) ***  **Absent**  **Present (hyaline)** | 20 (28.2%)  51 (71.8%) |
| **Amyloid deposits (n=40) ***  **Absent**  **Present** | 14 (35%)  26 (65%) |
| **Invasion (vascular and/or capsular) (n=25) ***  **Absent**  **Present** | 4 (16%)  21 (84%) |
| **Extrathyroidal extension (n=21) ***  **Absent**  **Present** | 7 (33.3%)  14 (66.7%) |
| **Metastasis (lymph node and/or distant) (n=29) ***  **Absent**  **Present** | 11 (37.9%)  18 (62.1%) |
| ***RET* mutation (n=75)**  **Absent**  **Present**  **- *germinative***  **- *somatic*** | 37 (49.3%)  38 (50.7%)  *7 (18.4%)*  *31 (81.6%)* |
| ***RAS* mutation (n=75)**  **Absent**  **Present** | 66 (88%)  9 (12%) |
| **PTEN nucl. % cellular expression**  **0%**  **0-25%**  **25-50%**  **50-75%**  **75-100%** | 30 (40%)  18 (24%)  9 (12%)  8 (10.7%)  10 (13.3%) |
| **PTEN citopl. % cellular expression**  **0%**  **0-25%**  **25-50%**  **50-75%**  **75-100%** | 4 (5.3%)  6 (8%)  4 (5.3%)  9 (12%)  52 (5%) |
| **pS6 % cell. expression**  **0%**  **0-25%**  **25-50%**  **50-75%**  **75-100%** | 11 (14.7%)  8 (10.7%)  -  10 (13.3%)  46 (61.3%) |
| **OPN % cell. expression***  **<5%**  **5-25%**  **25-50%**  **50-75%**  **75-100%** | 13 (17.3%)  3 (4%)  14 (18.7%)  8 (10.7%)  34 (45.3%) |

*Some clinicopathological/immunohistochemical features were not available for all the cases
