# Supplementary figures and images for: Calcitonin receptor expression in medullary thyroid carcinoma
Source: PeerJ. 2017 Sep 13;5:e3778. doi: 10.7717/peerj.3778 (PMC5600720; doi:10.7717/peerj.3778)

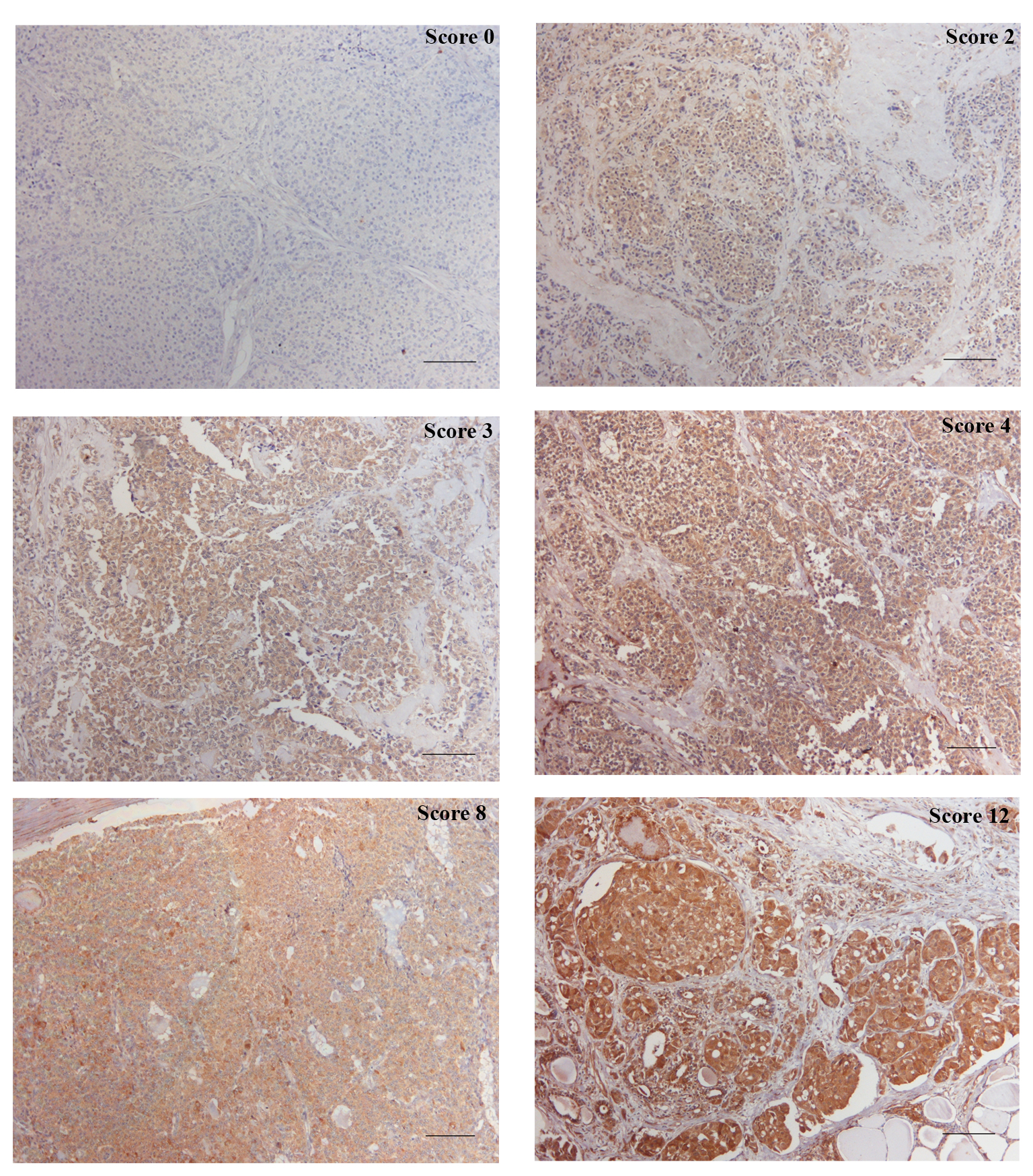

Supplement: Supplemental Information 2 — (A) Score 0 absence of staining. (B) Score 2 (extent 25–50%, intensity 1+). (C) Score 3 (extent 50–75%, intensity 1+). (D) Score 4 (extent 25–50%, intensity 2+). (E) Score 8 (extent 75–100%, intensity 2+). (F) (extent 100% intensity 3+). Photomic rographs were taken at 10x magnification. Bar 100μm. [file peerj-05-3778-s002.jpg]
